# Insegurança alimentar em comunidades quilombolas do Marajó, Amazônia brasileira: prevalência e fatores associados

**DOI:** 10.1590/0102-311XPT171825

**Published:** 2026-07-27

**Authors:** Regiane Padilha dos Santos, Victória Brioso Tavares, Maria Fernanda Brito de Matos, Socorro Castelo-Branco

**Affiliations:** 1 Universidade Federal do Pará, Belém, Brasil.

**Keywords:** Quilombolas, Segurança Alimentar e Nutricional, População Negra, Quilombola Communities, Food and Nutrition Security, Black People, Quilombola, Seguridad Alimentaria y Nutricional, Población Negra

## Abstract

O objetivo deste estudo foi determinar a prevalência de insegurança alimentar de comunidades quilombolas marajoaras e os fatores associados. Estudo transversal de base comunitária e abordagem familiar com 434 famílias, localizadas no Município de Salvaterra, Ilha de Marajó, Pará, Brasil, de junho a julho de 2021. Realizou-se inquérito domiciliar com questionário padronizado sobre dados sociodemográficos e o questionário da *Escala Brasileira de Insegurança Alimentar*. Para analisar os fatores associados foi utilizada a regressão logística multivariada. A maioria das famílias (88,9%) quilombolas estava em insegurança alimentar, 33,1% (n = 144) em insegurança grave, 21,4% (n = 93) em insegurança moderada e 34,3% (n = 149) em insegurança leve. O modelo de regressão mostrou que não receber aposentadoria dobrou a chance de insegurança alimentar leve e multiplica 5 vezes a insegurança moderada+grave. A renda familiar *per capita* de menos de 1/5 ou até 1/3 do salário mínimo elevou em 9 e 4 vezes a chance de insegurança moderada+grave, respectivamente. Famílias na classificação econômica B/C tiveram 21% menos chances de apresentar insegurança moderada+grave em relação às com classificação D/E. A insegurança alimentar apresentou alta prevalência em famílias quilombolas, preponderando insegurança moderada+grave. A renda familiar e seus componentes foram o principal fator associado à insegurança alimentar.

## Abstract (en)

This study aimed to determine the prevalence of food insecurity in Marajoara *quilombola* communities and its associated factors. A cross-sectional, community-based, family-approach study was carried out with 434 families in Salvaterra, Marajó Island, Pará, Brazil, from June to July 2021. A household survey was carried out with a standardized questionnaire on sociodemographic data and the *Brazilian Food Insecurity Scale*. A multivariate logistic regression was used to analyze associated factors. Most *quilombola* families (88.9%) lied in food insecurity, 33.1% (n = 144) of whom in severe insecurity; 21.4% (n = 93), in moderate insecurity; and 34.3% (n = 149), in mild insecurity. The regression model showed that receiving no pension doubles the chance of mild food insecurity and multiplies moderate+severe insecurity by 5 times. Per capita family income below 1/5 or even 1/3 of the minimum wage increase the chance of moderate+severe insecurity by 9 and 9 times, respectively. Households in the B/C economic classification were 21% less likely to have moderate+severe insecurity than those with the D/E classification. Food insecurity had a high prevalence in quilombola families, with a preponderance of moderate+severe insecurity. Family income and its components configured the main factors associated with food insecurity.

## Abstract (es)

El objetivo de este estudio fue determinar la prevalencia de la inseguridad alimentaria en las comunidades quilombolas de Marajó y los factores asociados. Se trata de un estudio transversal, de base comunitaria y con un enfoque familiar, con 434 familias ubicadas en el municipio de Salvaterra, Isla de Marajó, Pará, Brasil, de junio a julio del 2021. Se realizó una encuesta domiciliaria utilizando un cuestionario estandarizado sobre datos sociodemográficos y el cuestionario de la *Escala Brasileña de Inseguridad Alimentaria*. Se utilizó la regresión logística multivariante para analizar los factores asociados. La mayoría de las familias quilombolas (88,9%) sufría inseguridad alimentaria, de las cuales el 33,1% (n = 144) experimentaba inseguridad severa, el 21,4% (n = 93) inseguridad moderada y el 34,3% (n = 149) inseguridad leve. El modelo de regresión demostró que no recibir jubilación duplicaba la probabilidad de sufrir inseguridad alimentaria leve y multiplicaba por 5 la probabilidad de sufrir inseguridad moderada+severa. Un ingreso familiar per cápita inferior a 1/5 o hasta 1/3 del salario mínimo aumentó la probabilidad de inseguridad alimentaria moderada+severa en 9 y 4 veces, respectivamente. Las familias de la categoría económica B/C tuvieron un 21% menos de probabilidades de experimentar inseguridad moderada+severa en comparación con las de la categoría D/E. La inseguridad alimentaria tuvo una alta prevalencia entre las familias quilombolas, predominando la inseguridad de moderada+severa. Los ingresos familiares y sus componentes fueron el principal factor asociado a la inseguridad alimentaria.

## Introdução

A insegurança alimentar corresponde à dificuldade ou incapacidade de obter alimentação pela ausência de recursos para a aquisição de alimentos e inconsistência no acesso à alimentação, sendo considerada pela Organização das Nações Unidas como um desafio a ser enfrentado até 2030, para o desenvolvimento sustentável ^1^ ^,^ ^2^.

Em 2021, em decorrência da pandemia de COVID-19, o Brasil retornou ao “Mapa da Fome” após sete anos e em 2022 cerca de um quarto da população apresentava algum grau de insegurança alimentar ^3^, no período pós-pico da pandemia apresentou redução contínua dos indicadores, resultando na saída deste mapa em 2025 ^4^, ano em que o país alcançou 213 milhões de habitantes.

Nesse período, as desigualdades sociais impactaram diretamente a insegurança alimentar em populações negras africanas, caribenhas e asiáticas ^5^. No *I Inquérito Nacional sobre Insegurança Alimentar no Contexto da Pandemia da COVID-19 no Brasil* - VIGISAN (2020), 55,2% das famílias estavam em algum grau de insegurança alimentar no país, 63,2% na Região Norte ^6^. No II VIGISAN (2021-2022), a insegurança alimentar foi de 58,7% no país, 87,3% na Região Norte e chegando a 91,3% no Estado do Pará ^3^. Essa piora está relacionada à redução de empregos e ao empobrecimento da população ^3^.

No Brasil, existem mais de três mil comunidades remanescentes de quilombos. Elas existem desde o período colonial e foram constituídas em resistência ao sistema escravista ^7^ ^,^ ^8^. A discussão sobre insegurança alimentar em comunidades quilombolas é desafiadora, pois transcorre por um processo histórico definido pelo enfrentamento das ameaças territoriais e luta por políticas públicas concretas, que interferem no acesso a alimentos tradicionais ^9^; todos esses aspectos são reflexos do racismo ^10^.

A primeira pesquisa sobre insegurança alimentar em comunidades quilombolas (2006), realizada em 60 comunidades de diferentes regiões do país, mostrou que 7,5% das crianças menores de 11 anos consumiam menos de três refeições diárias ^11^. Em diversas comunidades quilombolas do país, a insegurança alimentar atinge mais de 50% das famílias somando-se às precárias condições de vida observadas nos domicílios ^9^ ^,^ ^12^ ^,^ ^13^ ^,^ ^14^ ^,^ ^15^.

A proteção social dessas comunidades sofreu com o desmonte de ações de segurança alimentar, como a extinção do Ministério do Desenvolvimento Social e Combate à Fome (MDS), a redução do orçamento para programas de distribuição de alimentos entre 2016-2019 ^16^ e a diminuição dos recursos do Bolsa Família, o que reforçou a invisibilização da população quilombola. Associado ao isolamento geográfico acentuado pela COVID-19, esse quadro contribuiu para a manutenção da pobreza ^17^.

É nesse cenário que se inserem as comunidades quilombolas do arquipélago de Marajó (Pará). Dessa forma, o objetivo deste estudo foi determinar a prevalência de insegurança alimentar nas comunidades quilombolas marajoaras e fatores associados em 2021, período em que seria possível observar o resultado do desmonte de políticas públicas de proteção social.

## Materiais e métodos

Estudo transversal de base comunitária com abordagem familiar, desenvolvido nos meses de junho a julho de 2021 em 14 comunidades quilombolas, localizadas no Município de Salvaterra, Ilha de Marajó: Campina/Vila União, Bacabal, Santa Luzia, Rosário, Boa Vista, Deus Ajude, Bairro Alto, Pau Furado, São Benedito da Ponta, Siricari, Providência, Mangueira, Salvá e Paixão, todas reconhecidas pela Fundação Cultural Palmares, instituição responsável por certificar as comunidades quilombolas ancestrais ^18^.

### População e amostra do estudo

Do total de 751 famílias, conforme dados disponibilizados pela Secretaria Municipal de Saúde de Salvaterra com base nas famílias cadastradas no e-SUS território (https://www.gov.br/pt-br/apps/e-sus-territorio), em colaboração com as comunidades, foi calculada amostra estratificada proporcional ao número total de famílias de cada comunidade. A amostra foi calculada com erro amostral de 6% e intervalo de 95% de confiança, totalizando 237 domicílios com acréscimo de 20% para cobrir possíveis perdas; chegou-se à amostra mínima de 284 famílias. Em cada domicílio, o responsável pela família, identificado por seus membros, foi o respondente da *Escala Brasileira de Insegurança Alimentar* (EBIA) ^19^. Em caso de recusa ou ausência de moradores após três tentativas de visita, foi realizado novo sorteio na mesma comunidade.

### Coleta de dados

O inquérito domiciliar para a aplicação do questionário padronizado foi realizado no período de junho a julho de 2021, por entrevistadores previamente treinados e supervisionados. Para a aplicação do questionário foi utilizado o programa DataScope (https://datascope.io/pt/) que permite a coleta de dados *offline* por meio de seu aplicativo disponibilizado no celular. Em campo, os esclarecimentos, a localização das famílias selecionadas e o convite para a participação no estudo ocorreu com o auxílio dos agentes comunitários de saúde ou de líderes comunitários.

O questionário padronizado foi constituído por dados pessoais do respondente da família: data de nascimento; sexo (masculino e feminino); escolaridade, última série finalizada (primeiro grau completo, 2º grau incompleto, 2º grau completo, universitário incompleto, universitário completo, pós-graduação).

A renda familiar (em reais) foi dividida em: sem renda, menos de 1.045,00, 1.045,00, entre 1.046,00 e 1.567,50, entre 1.568,00 e 2.090,00, entre 2.091.00 e 3.135,00, entre 3.136,00 e 4.180,00, entre 4.181,00 e 5.225,00, e acima de 5.225,00.

A situação econômica foi baseada na Classificação Econômica Brasil (2020) ^20^ e considerou a quantidade de posse de bens (vídeo-cassete/DVD, automóvel, máquina de lavar louça, micro-ondas, secadora de roupa, moto, máquina de lavar roupa, geladeira simples, geladeira dúplex, *freezer*, computador, pagar empregada doméstica, e casa com banheiro); acesso aos serviços públicos (rua pavimentada e água encanada no domicílio); auxílio do governo (Bolsa Família, aposentadoria, pensão, auxílio defeso, auxílio emergencial); e grau de instrução do chefe da família.

As condições de moradia foram constituídas pela situação do domicílio (alugado, próprio já pago, próprio ainda pagando, próprio em área de ocupação, propriedade rural, cedido por pessoa/empresa); tipo de material da casa (tijolo, madeira, barro, mista, outro material); destino do esgoto (rio ou igarapé, rede de esgoto, fossa); tratamento da água para beber (hipoclorito, fervura, filtro, outro tipo); e abastecimento de água (rio ou igarapé, poço aberto, mineral comprada, poço fechado com bomba, companhia de água).

A insegurança alimentar foi mensurada de acordo com a EBIA composta por 14 perguntas, para as quais se admite resposta dicotômica (sim ou não), compreendendo o período de três meses anteriores à entrevista. Cada resposta afirmativa (“sim”) equivale a um ponto. A soma total determina a classificação do domicílio que varia de acordo com a presença ou ausência de menores de 18 anos, respectivamente: segurança alimentar (0), insegurança alimentar leve (1-5; 1-3), insegurança alimentar moderada (6-9; 4-5) e insegurança alimentar grave (10-14; 6-8) ^19^.

### Variáveis do estudo

Neste estudo a variável de desfecho foi a insegurança alimentar, analisada em três categorias com o agrupamento das categorias insegurança alimentar moderada e insegurança alimentar grave. As covariáveis de estudo foram: idade, anos de escolaridade, número de moradores na casa, número de moradores menores de 18 anos, sexo, classificação econômica, renda *per capita*, Bolsa Família, aposentadoria, auxílio COVID-19, situação do domicílio, tipo de material da casa, destino do esgoto, tratamento da água para beber e abastecimento de água.

### Análise estatística

Inicialmente, as diferenças entre as categorias das variáveis sociodemográficas e de moradia foram testadas pelo qui-quadrado de Pearson ou exato de Fisher, para as variáveis categóricas e para as variáveis contínuas pelo teste Kruskal-Wallis para amostras independentes, pois o teste Kolmogorov-Smirnov mostrou distribuição não paramétrica para todas as variáveis contínuas. Foram construídos modelos de regressão logística multinomial com abordagem *enter*. O modelo inicial partiu da inclusão das variáveis que na análise univariada prévia apresentaram valor de p < 0,20 na categoria insegurança alimentar moderada+grave ^21^. A escolha do conjunto de variáveis do modelo final baseou-se na ausência de multicolinearidade entre as variáveis, considerando o fator de inflação de variância (VIF, de *variance inflation factor*) menor que 10 e a tolerância maior que 0,10, e considerando estatisticamente significante os valores de p < 0,05. A análise estatística foi realizada no software IBM SPSS Statistics 22.0 (https://www.ibm.com/).

### Aspectos éticos

Este estudo integra o projeto *Katuana Quilombola* [Katuana significa “gente boa” em Tupi-Guarani], que foi aprovado pelo Comitê de Ética em Pesquisa da Universidade Federal do Pará (CAAE 29865219.8.0000.0018), e segue a *Resolução nº 466/2012* do Conselho Nacional de Saúde. Todos os participantes receberam explicação verbal e escrita, por meio do Termo de Consentimento Livre e Esclarecido (TCLE) sobre o objetivo do estudo, procedimentos a serem realizados e riscos e benefícios da participação. Os que aceitaram participar assinaram o TCLE, quando analfabeto o consentimento foi mediante impressão digital com testemunha. As comunidades participantes aceitaram a realização desta pesquisa de campo, firmado pelo termo de aceite.

## Resultados

Participaram deste estudo 434 famílias, das quais 79,3% eram chefiadas por mulheres, com mediana de idade de 42 anos e seis anos completos de estudo. A condição de insegurança alimentar foi identificada em 88,9% das famílias quilombolas.

Os grupos de insegurança alimentar leve e insegurança alimentar moderada+grave apresentaram chefes de família mais jovens, com menos anos completos de estudo e com maior número de moradores no mesmo domicílio, além de maior proporção de menores de 18 anos (Tabela 1).

**Tabela 1:** Caracterização das condições socioeconômicas e habitacionais de acordo com a classificação de (in)segurança alimentar em comunidades quilombolas. Município de Salvaterra, Ilha de Marajó, Pará, Brasil, 2021.

Variáveis	(In)segurança alimentar [n (%)]			Valor de p
	Segurança (n = 48)	Leve (n = 149)	Moderada+grave (n = 237)	
Idade [média (DP)] *	54,0 (44,5-61,0)	39,0 (36,0-42,0)	44,5 (39,0-48,0)	0,001 **
Anos de estudo [média (DP)] *	8,0 (6,5-11,0)	7,0 (6,0-8,0)	5,0 (5,0-6,0)	0,012 **
Sexo *				0,199 ***
Masculino	14 (29,2)	33 (22,1)	43 (18,1)	
Feminino	34 (70,8)	116 (77,9)	194 (81,9)	
Número de moradores na casa [média (DP)]	3 (2-4)	4 (3-4)	4 (4-4)	0,005 **
Presença de moradores menores de 18 anos				< 0,001 ***
Não	27 (56,3)	36 (24,2)	71 (30,0)	
Sim	21 (43,8)	113 (75,8)	166 (70,0)	
Classificação econômica				< 0,001 ^#^
B/C	11 (22,9)	12 (8,1)	9 (3,8)	
D/E	37 (77,1)	137 (91,9)	228 (96,2)	
Renda *per capita* (R$)				< 0,001 ***
0,00-194,46	7 (14,6)	63 (42,3)	147 (62,0)	
194,47-366,33	7 (14,6)	31 (20,8)	47 (19,8)	
Acima de 366,33	34 (70,8)	55 (36,9)	43 (18,1)	
Bolsa Família				0,051 ^#^
Não	20 (41,7)	46 (30,9)	59 (24,9)	
Sim	28 (58,3)	103 (69,1)	178 (75,1)	
Aposentadoria				< 0,001 ***
Não	21 (43,8)	110 (73,8)	186 (78,5)	
Sim	27 (56,3)	39 (26,2)	51 (21,5)	
Auxílio COVID-19				0,021 ***
Ambos	15 (31,3)	42 (28,2)	74 (31,2)	
Apenas o primeiro	14 (29,2)	55 (36,9)	110 (46,4)	
Nenhum	19 (39,6)	52 (34,9)	53 (22,4)	
Situação do domicílio				0,335 ***
Não próprio	8 (16,7)	23 (47,9)	17 (35,4)	
Próprio	40 (10,4)	219 (56,7)	127 (32,9)	
Tipo de material da casa				0,478 ^#^
Alvenaria	48 (100,0)	232 (95,9)	139 (96,5)	
Madeira ou mista	0 (0,0)	10 (4,1)	5 (3,5)	
Destino do esgoto				0,305 ***
Rio ou igarapé	24 (10,0)	130 (53,9)	87 (36,1)	
Fossa/Rede de esgoto	24 (12,4)	112 (58,0)	57 (29,5)	
Tratamento da água para beber				0,312 ***
Sem tratamento	17 (9,1)	101 (54,3)	68 (36,6)	
Hipoclorito/Filtração/Fervura	31 (12,5)	141 (56,9)	76 (30,6)	
Abastecimento de água				0,292 ***
Poço	42 (11,3)	212 (57)	118 (31,7)	
Companhia de água	6 (9,7)	30 (48,4)	26 (41,9)	

DP: desvio padrão.

* Referente ao indivíduo respondente da entrevista;

** Teste Kruskal-Wallis de amostras independentes;

*** Teste qui-quadrado de Pearson;

^#^ Teste de Fisher.

Observou-se predomínio geral de famílias na classificação econômica D/E, porém as frequências foram maiores entre os grupos de insegurança leve (91,9%) e moderada+grave (96,2%). Mais da metade (62%) das famílias em insegurança alimentar moderada+grave não tinham nenhuma renda ou sobreviviam com até R$ 194,46. Entre as famílias em insegurança alimentar moderada+grave, 78,5% não contavam com a aposentadoria como fonte de renda; este resultado correspondeu a 73,8% das famílias em insegurança alimentar leve (Tabela 1).

A maior parcela das famílias recebeu o auxílio COVID-19 disponibilizado pelo Governo Federal durante a pandemia. Entretanto, 46,4% em insegurança alimentar moderada+grave e 36,9% em insegurança alimentar leve receberam apenas a primeira parcela do benefício (Tabela 1). Cerca de 28,6% das famílias não receberam o auxílio.

A maior parte das famílias foi beneficiada pelo programa Bolsa Família, 75,1% em insegurança alimentar moderada+grave e 69,1% em insegurança alimentar leve (Tabela 1), no entanto, mais de 27,2% delas em insegurança alimentar não recebiam este auxílio.

A maioria das famílias morava em domicílio próprio (88,9%) com construção em alvenaria (96,5%). O abastecimento da água era realizado por poço fechado ou aberto em 85,7% das residências, sendo que mais da metade realizava o tratamento da água com hipoclorito, fervura ou filtragem. Além disso, a maioria (87,3%) informou que o destino do esgoto do banheiro era em fossa/rede pública. Nenhum desses fatores apresentou diferença estatisticamente significante na comparação entre as categorias de insegurança alimentar.

Todas as variáveis foram utilizadas para a análise de regressão univariada, nenhuma apresentou multicolinearidade. No grupo insegurança alimentar moderada+grave, todas as variáveis analisadas (idade, anos de estudo, número de moradores na casa, presença de moradores menores de 18 anos, sexo, classificação econômica, renda *per capita*, Bolsa Família, aposentadoria, auxílio COVID-19) apresentaram valor de p < 0,20. Dessa forma, todas essas variáveis foram incluídas para a construção do modelo (Tabela 2). O modelo final da regressão multinomial (Tabela 2) apontou: famílias que não recebiam aposentadoria estavam associadas ao dobro de chances de apresentar insegurança alimentar leve e 5 vezes mais chances de insegurança alimentar moderada+grave.

**Tabela 2:** Modelo final da análise multinomial para insegurança alimentar leve e moderada+grave, tendo como categoria de referência a segurança alimentar. Comunidades quilombolas de Salvaterra, Ilha de Marajó, Pará, Brasil, 2021.

Variáveis	Insegurança alimentar leve			Insegurança alimentar moderada+grave
	OR	IC95%	OR	IC95%
Idade	0,98	0,94-1,01	1,02	0,98-1,05
Anos de estudo	0,97	0,88-1,07	0,92	0,83-1,02
Número de moradores na casa	1,12	0,80-1,56	1,17	0,84-1,63
Presença de moradores menores de 18 anos	0,98	0,59-1,62	0,90	0,55-1,48
Sexo				
Homem	0,74	0,33-1,67	0,50	0,22-1,14
Mulher	Referência	Referência	Referência	Referência
Classificação econômica				
B/C	0,33	0,10-1,04	0,21	0,06-0,76 *
D/E	Referência	Referência	Referência	Referência
Renda *per capita* (R$)				
0,00-194,46	2,63	0,91-7,60	9,79	3,41-28,11 *
194,47-366,33	2,33	0,82-6,64	4,70	1,66-13,34 *
Acima de 366,33	Referência	Referência	Referência	Referência
Bolsa Família				
Não	1,67	0,67-4,17	1,63	0,65-4,09
Sim	Referência	Referência	Referência	Referência
Aposentadoria				
Não	2,90	1,02-8,21 *	5,92	2,02-1,74 *
Sim	Referência	Referência	Referência	Referência
Auxílio COVID-19				
Ambos	0,47	0,16-1,36	1,00	0,35-2,87
Apenas o primeiro	0,86	0,33-2,28	2,09	0,79-5,55
Nenhum	Referência	Referência	Referência	Referência

IC95%: intervalo de 95% de confiança; OR: *odds ratio*.

* Valor de p < 0,05.

Ter renda familiar *per capita* de até R$ 194,46 esteve associada com 9 vezes mais chances de insegurança alimentar moderada+grave, e renda entre R$ 194,47 e R$ 366,33 esteve associada a até 4 vezes mais chances. Também para a categoria de insegurança alimentar moderada+grave, fazer parte das classes econômicas B/C mostrou efeito protetor de 21%.

## Discussão

O estudo demonstrou que a insegurança alimentar afeta as famílias quilombolas em Salvaterra, com uma prevalência de 88,9%, superando a média nacional (46,5%) ^16^ durante a pandemia em 2020 (63,2%) ^6^ e em 2021-2022 (87,3%) ^3^. Esses resultados foram semelhantes às proporções encontradas em diferentes regiões do Brasil em estudos pré-pandemia: 70% (2008) e 85,1% (2016) no Tocantins ^15^ ^,^ ^22^, 75,2% em Goiás ^23^, 79,9% no Maranhão ^13^, 83,3% em Minas Gerais ^11^ e 64,9% na Bahia ^13^, demonstrando que essa já era uma realidade consolidada para essas comunidades.

O presente estudo foi conduzido após a primeira onda de COVID-19, quando as comunidades já haviam recebido a vacina por serem integrantes de grupo prioritário. A insegurança alimentar nas famílias quilombolas também poderia ter sido agravada pela pandemia de COVID-19. No entanto, não há evidências que indiquem que a situação de insegurança alimentar observada nessas comunidades fosse diferente do período anterior à pandemia, tendo em vista a gravidade do cenário encontrado e a semelhança com outros estudos pré-pandemia em comunidades quilombolas ^13^ ^,^ ^14^ ^,^ ^15^ ^,^ ^22^ ^,^ ^23^, bem como com os resultados do II VIGISAN (2021-2022) no Pará ^3^. Assim, a emergência sanitária da COVID-19 evidenciou, mas não necessariamente modificou, a condição de vulnerabilidade alimentar já existente.

Nas comunidades remanescentes quilombolas do arquipélago de Marajó, foram notificados 75 casos de COVID-19 no período de março a setembro de 2020, distribuídos entre sete comunidades quilombolas do Município de Salvaterra. Algumas comunidades não apresentaram casos notificados, o que pode ser devido à baixa oferta dos serviços de saúde, refletindo em subdiagnóstico ou ao isolamento social, que reduziu a exposição à doença ^24^ ^,^ ^25^.

Importantes estratégias de combate à pandemia foram organizadas pela comunidade: barreiras sanitárias espontâneas, cooperação para compartilhar alimentos que antes da pandemia eram comercializados nas feiras livres municipais. Organizações sociais quilombolas e a prefeitura de Salvaterra forneceram cestas básicas ^26^. A participação do Governo Federal, de extrema direita, foi insuficiente para a proteção alimentar, considerando que 27,3% das famílias em insegurança alimentar não receberam auxílio COVID-19.

As comunidades quilombolas constituem uma estratégia de resistência da população negra, preservando raízes históricas, culturais e projetos de futuro ^10^, o que se expressa na luta pela titulação dos territórios. Abdias Nascimento ^27^ denominou essa mobilização de quilombismo, entendido como prática coletiva de resistência física e cultural, em que a população negra assume o comando de sua própria história. No quilombismo se resiste ao racismo, que invisibiliza culturas, artes, línguas e religiões de origem africana.

Nos territórios quilombolas a agricultura de subsistência contribui para a preservação ambiental, embora o direito à terra seja constantemente ameaçado. O Brasil concentra os maiores latifúndios do mundo, resultado da Lei de Terras de 1850, que dificultou o acesso à pequena propriedade e favoreceu a elite latifundiária branca ^28^. Segundo Bento ^28^, esse marco contribuiu para perpetuar uma estrutura racista em que esse grupo segue ocupando posições de poder econômico, enquanto a população negra permanece sub-representada, com maiores índices de pobreza, desemprego e limitado acesso à terra.

Isso se reflete na história e economia da Região Marajoara, onde a grilagem é processo contínuo desde o século XVIII, pela hegemonia de um pequeno grupo de latifundiários das fazendas de cana-de-açúcar e de gado ^29^. Esse movimento se intensificou com a chegada de produtores de arroz a Salvaterra que, em articulação com órgãos estaduais, passaram a interferir na produção tradicional e nas formas de subsistência das comunidades ^30^.

A expansão da monocultura do arroz nos territórios quilombolas marajoaras substitui a agricultura familiar por uma lógica produtiva capitalista dos latifúndios, contaminando terra e água com agrotóxicos. Esse processo ocorre com a anuência do Estado, na ausência do aval dos órgãos ambientais e contra a vontade das comunidades. Assim, evidencia-se que os quilombos são territórios racializados, onde o modo de vida negro é alterado por agentes do poder econômico que operam o pacto da branquitude, conforme conceituado por Cida Bento ^28^, perpetuando sobre eles o racismo ambiental ^31^ ^,^ ^32^.

Essas atividades prejudiciais ao meio ambiente impactam negativamente a disponibilidade de recursos alimentares, como água potável, alimentos seguros e ambientes saudáveis. As mudanças ambientais e culturais, ocorridas por introdução de monoculturas como a do arroz, e pela grilagem de terras, resultam na escassez ou ausência de recursos naturais como rios, lagos e manguezais, levando as comunidades a dependerem de recursos financeiros oriundos, em geral, de programas sociais. Além disso, passam a depender também da venda de sua força de trabalho para adquirir alimentos, o que contribui para o agravamento das condições de sobrevivência ^6^ ^,^ ^13^ ^,^ ^33^.

Considerando que a população negra, e dentro desta a população quilombola, é historicamente impactada pela baixa renda no Brasil, embora a maioria das comunidades quilombolas esteja localizada em áreas rurais, pode-se perceber que sua localização não explica, por si só, a alta taxa de insegurança alimentar ^22^. Estudos da população geral indicam que os principais fatores relacionados à insegurança alimentar estão associados aos determinantes socioeconômicos como renda *per capita* e escolaridade ^22^.

Neste estudo, verificou-se um baixo número de anos completos de escolaridade (5 anos) entre os respondentes cujas famílias se encontravam em insegurança alimentar moderada+grave, achado semelhante ao de outros estudos com comunidades quilombolas no Brasil ^13^ ^,^ ^23^ ^,^ ^34^ ^,^ ^35^. Apesar disso, a baixa escolaridade do respondente não foi associada a maiores chances de insegurança alimentar. Dessa forma, a baixa renda, relacionada à manutenção da desigualdade e à persistência do empobrecimento da população negra, conforme a herança racista destacada por Bento ^28^, mostrou ser o principal fator associado à insegurança alimentar nessa população.

A associação entre segurança alimentar e renda é documentada na literatura científica ^36^ ^,^ ^37^ ^,^ ^38^ ^,^ ^39^. Neste estudo, as chances de insegurança alimentar moderada+grave entre famílias com renda *per capita* nas faixas de R$ 0,00 a R$ 194,46 e de R$ 194,47 a R$ 366,33 são maiores, o que converge com a situação de outras comunidades quilombolas ^40^ ^,^ ^41^ e assemelha-se à proporção nacional identificada no VIGISAN de 2021, em que 85,3% das residências no Brasil tinham renda de 1/4 do salário mínimo *per capita* ^6^.

Outros trabalhos mostraram que as chances de insegurança alimentar moderada a grave aumentavam de maneira inversamente proporcional ao valor da renda *per capita* ^21^ ^,^ ^42^ ^,^ ^43^. Cherol et al. ^36^ demonstraram que, desde 2011, os domicílios quilombolas com renda familiar entre 1/2 e 1 salário mínimo apresentaram 3 vezes mais chances de estar em condição de insegurança alimentar grave ou moderada, em comparação com domicílios que tinham renda familiar maior que 1 salário mínimo vigente na época.

A análise multinomial revelou que a ausência de renda, proveniente da aposentadoria, também esteve associada ao dobro de chances de a família apresentar insegurança alimentar leve e 5 vezes mais chances de estar em insegurança alimentar moderada+grave, em comparação às famílias em segurança alimentar. Em 2021, dados nacionais corroboram essa tendência, uma vez que entre os 21,2% com algum membro da família recebendo aposentadoria, 58,2% destes estavam em segurança alimentar ^6^. Dado esse que reforça a relevância da aposentadoria como fator de proteção para a insegurança alimentar.

Rosa et al. ^44^ sugerem que a maior segurança alimentar, entre famílias compostas por adultos e idosos, está relacionada à contribuição das aposentadorias na renda familiar. Nas comunidades quilombolas de Salvaterra, observou-se papel semelhante, com esse benefício compondo a renda das famílias em segurança alimentar. Esse cenário evidencia a vulnerabilidade do mercado de trabalho para os trabalhadores ativos e a consequente dependência da renda de proteção social.

A classificação econômica visa a identificar grupos socioeconômicos pela capacidade de consumo. Neste estudo, fazer parte das classes econômicas B/C mostrou efeito protetor de 21% para a categoria de insegurança alimentar moderada+grave. Pesquisa realizada no Paraná, apontou que as classes D/E apresentaram 3,34 vezes mais chances de possuir insegurança alimentar moderada+grave em relação à classificação C ^43^.

Um estudo quilombola no Nordeste ^13^ mostrou que ser beneficiário do Bolsa Família e ter quatro residentes, ou mais, no domicílio, foram fatores associados à insegurança alimentar. Na população quilombola de Salvaterra, diferentemente do estudo nordestino, a ausência de recurso da aposentadoria para a família, classificação econômica D/E e a baixa renda *per capita* foram os fatores associados à insegurança alimentar.

O modelo conceitual de Lignani et al. ^37^, com base em estudos com populações não quilombolas, sugere que a renda tem uma relação direta com a insegurança alimentar e atua como mediador entre ela e os indicadores sociais. Embora o modelo forneça uma estrutura analítica das relações entre os fatores associados à insegurança alimentar, ele precisa ser considerado à luz das especificidades étnico-raciais para a população quilombola, em que a questão territorial e o racismo ambiental contribuem para essa relação entre renda e insegurança alimentar.

A propriedade do domicílio, o tipo de material da casa, assim como as condições sanitárias de água e esgoto não foram fatores associados à insegurança alimentar, resultados que divergem de outro estudo em comunidades quilombolas ^33^. Na população deste estudo, a maioria das famílias detinha a posse da moradia e participava de programa social para a construção da casa em alvenaria. Quanto à água e ao esgoto, as condições desfavoráveis englobavam toda a população de Salvaterra devido às dificuldades de territórios em áreas de ilha, além disso, o alto índice de vulnerabilidade social também é uma característica na região.

Os achados deste estudo devem ser interpretados considerando-se as limitações para estabelecer uma relação causal no estudo transversal, e diante da complexidade estrutural que caracteriza comunidades quilombolas, onde fatores históricos, raciais, territoriais e políticos podem não ser plenamente captados por variáveis mensuradas. Além disso, os inquéritos domiciliares com o uso de informantes secundários estão sujeitos a viés de relato pelo informante.

O desenvolvimento rural, a agricultura familiar e os programas de transferência de renda são fundamentais para fortalecer a autonomia quilombola ^45^. O Programa Brasil Quilombola (PBQ) integrou diversas políticas, como habitação, saneamento, saúde, educação e acesso à energia e à água, representando um marco institucional ^46^ ^,^ ^47^. Apesar de ter ampliado o número de titulações entre 2010 e 2014, há evidências de que o acesso aos recursos e benefícios foi limitado e pouco conhecido pelas comunidades ^47^ ^,^ ^48^.

Em 2023, o Programa Aquilomba Brasil retoma e atualiza essas diretrizes, estabelecendo 24 objetivos específicos e em seu segundo eixo prioriza a produção sustentável, a geração de renda, a soberania e a segurança alimentar. Além disso, prevê um comitê gestor para o monitoramento e a avaliação, apontando um avanço para sua implementação ^49^, pois, quando acessados, esses programas reduzem a probabilidade de insegurança alimentar, reforçam a autonomia comunitária e fortalecem o processo de aquilombamento ao oferecer alternativas à submissão aos latifúndios. Assim, contribuem para a resistência ao pacto da branquitude no meio rural marajoara, e são motores para a sobrevivência da agricultura familiar quilombola.

## Conclusão

Dentre a amostra de 434 famílias, a prevalência de insegurança alimentar nas comunidades quilombolas de Salvaterra foi de 88,9%, sendo que a maioria estava em insegurança moderada+grave. Do total, 33,18% estavam em insegurança grave, 21,43% em insegurança moderada e 34,33% das famílias em insegurança leve.

A alta prevalência de insegurança alimentar nas comunidades quilombolas de Salvaterra não será resolvida sem a mobilização dessa população, que historicamente foi protagonista no diálogo sobre a autoidentificação quilombola em suas comunidades e nos processos legais para a titulação da terra ^50^. Socialmente, a existência do pacto da branquitude e de como a pobreza e a desigualdade atingem a população quilombola, deve ser constantemente relembrada para que sua perpetuação seja questionada. Produções acadêmicas como este estudo servem para manter esses processos na memória e embasar com dados a necessidade de resistência da população quilombola, assim como de políticas públicas voltadas especificamente para a mesma.

A insegurança alimentar moderada+grave foi associada com a renda familiar *per capita* de até R$ 194,46 e ao não recebimento de aposentadoria por membros do grupo familiar, tendo como fator protetor a família estar inserida na categoria B/C da classificação econômica. A insegurança alimentar leve foi associada com o não recebimento de aposentadoria para a composição da renda familiar. Portanto, a renda e sua composição são os principais fatores associados à insegurança alimentar.

O Programa Aquilomba Brasil, do Governo Federal, que tem dentre seus objetivos a produção sustentável e geração de renda, soberania alimentar e segurança nutricional, pode ser uma estratégia para fortalecer o quilombismo, se as comunidades quilombolas tiverem acesso a essas informações, o que em muito depende da infraestrutura proporcionada pela gestão municipal e das organizações sociais quilombolas.

A situação de insegurança alimentar nas comunidades quilombolas de Salvaterra reflete a desigualdade estrutural e racista identificada neste estudo, pela baixa renda e vulnerabilidade socioeconômica. As iniciativas governamentais devem garantir a proteção social, sobretudo o acesso e a garantia ao território e a benefícios sociais, que constituem a base para a resistência contínua, autonomia e segurança alimentar dessas comunidades.

## Data Availability

Os dados de pesquisa estão disponíveis mediante solicitação à autora de correspondência.

## References

[1] Food and Agriculture Organization of the United Nations Hunger and food insecurity..

[2] Guterres A (2020). The sustainable development goals report 2020.

[3] Rede Brasileira de Pesquisa em Soberania e Segurança Alimentar e Nutricional II Inquérito Nacional sobre Insegurança Alimentar no Contexto da Pandemia da COVID-19 no Brasil (II VIGISAN): relatório final..

[4] Food and Agriculture Organization of the United Nations; International Fund for Agricultural Development; United Nations Children's Fund; World Food Program; World Health Organization The state of food security and nutrition in the world 2025..

[5] Dabone C, Mbagwu I, Muray M, Ubangha L, Kohoun B, Etowa E (2022). Global food insecurity and African, Caribbean, and Black (ACB) populations during the COVID-19 pandemic a rapid review. J Racial Ethn Health Disparities.

[6] Rede Brasileira de Pesquisa em Soberania e Segurança Alimentar e Nutricional Insegurança alimentar e COVID-19 no Brasil. Suplemento I: insegurança alimentar no estados..

[7] Fundação Cultural Palmares Certificação quilombola..

[8] Freitas DA, Caballero AD, Marques AS, Hernández CIV, Antunes SLNO (2011). Saúde e comunidades quilombolas uma revisão da literatura. Rev CEFAC.

[9] Afonso LFC, Correa NAF, Silva HP (2019). Segurança alimentar e nutricional em comunidades quilombolas no Brasil. Segur Aliment Nutr.

[10] Gonzalez L (2020). Por um feminismo afrolatinoamericano: ensaios, intervenções e diálogos.

[11] Secretaria de Avaliação e Gestão da Informação. Ministério do Desenvolvimento Social e Combate à Fome (2008). Políticas sociais e chamada nutricional quilombola: estudos sobre condições de vida nas comunidades e situação nutricional das crianças.

[12] Secretaria de Avaliação e Gestão da Informação, Ministério do Desenvolvimento Social e Combate à Fome (2014). Análise das condições de vida, segurança alimentar e nutricional e acesso a programas sociais em comunidades quilombolas tituladas..

[13] Silva EKP, Medeiros DS, Martins PC, Sousa LA, Lima GP, Rêgo MAS (2017). Insegurança alimentar em comunidades rurais no Nordeste brasileiro faz diferença ser quilombola?. Cad Saúde Pública.

[14] Ribeiro G, Morais FMO, Pinho L (2015). (In)Segurança alimentar de comunidade quilombola no norte de Minas Gerais.. Ciênc Cuid Saúde.

[15] Monego ET, Peixoto MRG, Cordeiro MM, Costa RM (2010). (In)segurança alimentar de comunidades quilombolas do Tocantins.. Segur Aliment Nutr.

[16] Carregosa MP, Sandes ACLL, Mendonça TS, Voci SM (2024). Panorama das políticas públicas de segurança alimentar e nutricional voltadas aos quilombolas após período de desmontes. Segur Aliment Nutr.

[17] Pinho L, Dias RL, Cruz LMA, Velloso NA (2015). Health conditions of quilombola community in the north of Minas Gerais. Revista de Pesquisa: Cuidado é Fundamental Online.

[18] Fundação Cultural Palmares (2021). Quadro geral de comunidades remanescentes de quilombos (CRQS).

[19] Segall-Corrêa AM, Marin-Leon L (2009). A segurança alimentar no Brasil proposição e usos da escala brasileira de medida da insegurança alimentar (EBIA) de 2003 a 2009. Segur Aliment Nutr.

[20] Associação Brasileira de Empresas de Pesquisa Alterações na aplicação do Critério Brasil, válidas a partir de 01/09/2020..

[21] Souza BFNJ, Marin-Leon L, Camargo DFM, Segall-Corrêa AM (2016). Demographic and socioeconomic conditions associated with food insecurity in households in Campinas, SP, Brazil. Rev Nutr.

[22] Instituto Brasileiro de Geografia e Estatística Pesquisa de Orçamentos Familiares 2017-2018: análise da segurança alimentar no Brasil..

[23] Cordeiro MM, Monego ET, Martins KA (2014). Overweight in Goiás' quilombola students and food insecurity in their families. Rev Nutr.

[24] Veiga Gonçalves N, De Melo JS, Kazumi da Trindade Noguchi S, Silva Machado A, Da Silva Martins A, Da Silva Peixoto MC (2021). COVID-19 in socially vulnerable quilombola populations in Salvaterra, Pará, Eastern Amazon, Brazil.. J Infect Dev Ctries.

[25] Sousa RF, Rodrigues IL, Pereira AA, Nogueira LM, Andrade EG, Pinheiro AK (2023). Health conditions and relationship with health services from Quilombola people's perspective. Esc Anna Nery Rev Enferm.

[26] Otoni de Souza L, Lopes Simonian LT, Pahoona Corbin H (2024). Superando a pandemia: o papel fundamental das lideranças nas comunidades quilombolas do Marajó.. Amazônia Investiga.

[27] Nascimento A (1980). O quilombismo: documentos de uma militância panafricanista.

[28] Bento C (2022). O pacto da branquitude.

[29] Carlos J, Meirelles DS (2014). Arroz no Marajó a impunidade do agronegócio. Inclusão Social.

[30] Cabral CL (2018). Conflitos territoriais nos quilombos da APA Arquipélago do Marajó/PA. Revista Eletrônica: Tempo Técnica Território.

[31] Pacheco T, Faustino C, Porto MF, Pacheco T, Leroy JP (2013). Injustiça ambiental e saúde no Brasil: o mapa de conflitos.

[32] Jesus V (2020). Racializando o olhar (sociológico) sobre a saúde ambiental em saneamento da população negra um continuum colonial chamado racismo ambiental. Saúde Soc.

[33] Maciel ES, Silva BKR, Schott E, Kato HCA, Quaresma FPR, Figueiredo FWS (2021). Insegurança alimentar em comunidades quilombolas. Segur Aliment Nutr.

[34] Guerra LDS, Espinosa MM, Bezerra ACD, Guimarães LV, Martins MSAS (2018). Desafios para a segurança alimentar e nutricional na Amazônia disponibilidade e consumo em domicílios com adolescentes. Ciênc Saúde Colet.

[35] Pinto AR, Borges JC, Novo MP, Pires PS (2014). Quilombos do Brasil segurança alimentar e nutricional e territórios titulados. Cadernos de Estudos Desenvolvimento Social em Debate.

[36] Cherol CCS, Ferreira AA, Salles-Costa R (2021). Desigualdades sociais e insegurança alimentar domiciliar em comunidades quilombolas no Brasil. Rev Nutr.

[37] Lignani JB, Palmeira PA, Antunes MML, Salles-Costa R (2020). Relação entre indicadores sociais e insegurança alimentar uma revisão sistemática. Rev Bras Epidemiol.

[38] Bezerra TA, Olinda RA, Peraza DF (2017). Food insecurity in Brazil in accordance with diferent socio-demographic scenarios. Ciênc Saúde Colet.

[39] Sousa LRM, Segall-Corrêa AM, Saint Ville A, Melgar-Quiñonez H (2019). Food security status in times of financial and political crisis in Brazil. Cad Saúde Pública.

[40] Aires JS, Martins MC, Joventino ES, Ximenes LB (2012). (In)Segurança alimentar em famílias de pré-escolares de uma zona rural do Ceará.. Acta Paul Enferm.

[41] Silva BMA, Silveira VNC, Padilha LL, Frota MTBA (2020). Situação de insegurança alimentar e nutricional em famílias quilombolas maranhenses. Demetra (Rio J.).

[42] Brito AP, Lima VN, Silva EGCM, Rêgo AS, Dias LPP, Silva JD (2020). Fatores associados à insegurança alimentar e nutricional em comunidade carente. Rev Bras Promoç Saúde.

[43] Anschau FR, Matsuo T, Segall-Corrêa AM (2012). Insegurança alimentar entre beneficiários de programas de transferência de renda. Rev Nutr.

[44] Rosa TEC, Mondini L, Gubert MB, Sato GS, Benício MHD'A (2012). Segurança alimentar em domicílios chefiados por idosos, Brasil.. Rev Bras Geriatr Gerontol.

[45] De Souza Cherol CC, Ferreira AA, Salles-Costa R (2021). Programas governamentais associados à insegurança alimentar em comunidades descendentes de negros escravizados no Brasil.. Public Health Nutr.

[46] Secretaria Especial de Políticas de Promoção da Igualdade Racial Programa Brasil Quilombola..

[47] Simonard P, César SB, Silva AS, Monteiro L (2020). Um estudo sobre a implementação do Programa Brasil Quilombola nos povoados Ribeira e Tabacaria, Alagoas.. Amazônica Revista de Antropologia.

[48] Barbosa MP, Braga LAM, Rodrigues CT Programa Brasil Quilombola: análise do processo de implementação..

[49] Brasil (2023). Decreto nº 11.447, de 21 de março de 2023. Institui o Programa Aquilomba Brasil e o seu Comitê Gestor.. Diário Oficial da União.

[50] Lima PM, Silveira FLA, Coelho LFC, Oliveira CO (2016). O desfile da raça: identidade e luta quilombola em Salvaterra, Ilha do Marajó, Pará.. Ambivalências.

